# Measuring and understanding labor exploitation on a sample of workers in the Costa Rican fishing industry

**DOI:** 10.1371/journal.pone.0341336

**Published:** 2026-02-06

**Authors:** Stephen Abeyta, Lauren N. Moton, Meredith Dank

**Affiliations:** Marron Institute of Urban Management, New York University, New York, New York, United States of America; International Islamic University Malaysia, MALAYSIA

## Abstract

Due to the challenging nature of surveying exploited workers, scholars are often limited in their ability to standardize data collection efforts. Using two samples of survey data from workers in the Costa Rican fishing industry, we compare latent classes of exploitative labor between a subset of individuals surveyed through a proportional probability sample (PPS) and Vincent-link-tracing sampling (VLTS). Results suggest there are some differences between latent classes across the differently sampled groups, with those in the PPS sample being more defined by severe forms of exploitation. Despite these differences, latent classes largely looked similar, with each group having a more highly exploited class and a more minimally exploited class. This study suggests that sampling techniques may affect the broader understanding of the issue, but most of the variation of exploitative experiences may lie in the characteristics of specific contexts or occupations.

## Introduction

There has been a marked increase in research on labor exploitation and trafficking in recent years, which has resulted in the identification of the fishing industry as a sector where exploitative working conditions are a significant issue [1–5]. Researchers have sought to understand the populations and dynamics within the industry that provide fertile ground for exploitative practices. Accurate and robust data are needed to assist in combating a pervasive issue that impacts approximately 128,000 fishers worldwide, according to a 2022 report by the International Labor Organization (ILO), Walk Free, and International Organization for Migration [6].

Despite the extensive documentation of labor trafficking and exploitation in the global fishing industry, approaches to properly measure this issue remain opaque due to various reasons. First, these offenses are typically conducted clandestinely, making them difficult to detect [7,8]. Additionally, victims of these abuses may not be aware of their rights or recognize themselves as victims, often blaming themselves for their situation [9]. Furthermore, there is no central system for reporting labor trafficking, and victims seldom come forward to report their circumstances [7]. The legal definition of labor trafficking is broad, varied, and complicated across different global contexts [7,8]. Finally, to conduct an empirical exploration into this issue, it can be challenging to ask participants to disclose their experiences of exploitation [7].

Stakeholders involved in various global anti-trafficking efforts require rigorous and representative data in order to effectively inform their responses to labor trafficking and exploitation within their countries and jurisdictions. A first step in this effort is to investigate how various methods impact how we understand the complex experiences of exploitation. We focus on Costa Rica, an under-researched geographic context, as it pertains to exploitative working conditions in the fishing industry. Before we explore two data collection methods, Probability Proportionate to Size (PPS) sample and Vincent Link Tracing Sample (VLTS), designed to capture the prevalence and unique experiences of hard-to-reach populations—like Costa Rican fishermen—we must first outline labor trafficking and exploitation, the Costa Rican fishing context, and measuring exploitative labor and the complexities therein.

## Literature review

### Labor trafficking and exploitation

The central concept in the current study is exploitative labor, which encompasses a broad range of harmful labor conditions that remain the focal point of global labor research [10]. Labor exploitation broadly entails situations in which workers are subjected to unfair, abusive, or illegal working conditions that violate labor laws or human rights standards. These situations often primarily benefit the employer and can include experiences of coercive recruitment, harmful employment practices and penalties (e.g., wage theft or excessive work hours without breaks), control over ones personal life (e.g., use of blackmail by an employer or coworker), being required to be available at all times for labor without adequate compensation, restrictions of physical or communicative freedom, and violence.

Labor exploitation focuses on the relationship between labor conditions and capital gain and lies on a spectrum of severity that may reach the legal threshold of labor trafficking if it involves force, fraud, or coercion [11]. ILO estimates 28 million people worldwide are affected [12] by labor trafficking which can include more severe forms of labor exploitation, like having to perform sex acts or labor to pay off debt or receive wages, imposition of debt without consent, or lose freedom of movement due to surveillance, experiencing isolation within the workplace, or losing the freedom to communicate with friends or family, experience coercive or deceptive recruitment, withholding pay or benefits, made to work without adequate compensation, confiscation of identity or travel documents, loss of freedom to communicate with loved ones, and physical or sexual violence against themselves or loved ones [13].

Ultimately, one of the historic challenges in the field of trafficking is the lack of a clear experiential definition and where it exists on the spectrum of exploitation [13]. While instances of exploitation may be violent in nature, violence in relation to work and the workplace is not inherently exploitative as it is not associated with the underlying cause of exchanging worker well-being for industrial gain.

Where there is additional ambiguity about exploitative labor and trafficking is the degree to which individual experiences are connected to workers versus the workplace. For instance, if an individual worker experiences the garnishment of their wages, physical abuse, emotional manipulation in the form of threats, and physical restraint within a specific room in a workplace, this individual would likely be classified as a victim of labor trafficking. However, if a worker experiences each of these individual exploitative events across different workplaces [14], it is unclear to what degree this worker has experienced complex exploitation or trafficking. Due to this ambiguity, this study largely focuses on the broad spectrum of exploitative experiences, including those that rise to the level of trafficking.

Underlying the complexity of labor exploitation is the manner by which context–at multiple levels–affects the forms of exploitation experienced by workers. Context here refers to the interwoven nexus of a specific time in which a specific worker is employed by a specific industry in a specific geographic location. These intricacies are important to consider in research on exploitative labor, as workers of different industries will be exposed to disparate occupational harms, as will workers of the same industry in differing positions or locations [15]. This relationship between a generally harmful working environment and context is exemplified in research on violence experienced by healthcare workers, as people with different roles within the same hospital experience varying forms of violence, and workers at hospitals in different cities also experience different forms of violence [15]. The effect of differing working contexts has similarly been recognized in trafficking literature, with scholars finding that exposure to trafficking and exploitative experiences may change based on country or region characteristics [14,16].

Important to consider are the contributors to exploitative working conditions in the fishing industry. Fishing is an important economic driver in many countries, and thus, the global demand for seafood creates conditions where abuse and exploitation are possible. The fishing industry has been empirically explored in relation to labor trafficking and exploitation, particularly in Southeast Asian contexts (e.g., Thailand, Taiwan, and the Philippines, see [3,4,17,18]. Exploited fishers report experiencing sickness, bodily harm, mental and sexual victimization, witnessing the deaths of fellow crew members, and being exposed to danger while working on ships in isolated areas of the ocean for extended periods of time. Fishers are required to work for extended periods for meager wages, and the job is demanding, hazardous, and arduous. Commercial fishing has one of the highest rates of work-related deaths among all industries worldwide [19].

Moreover, many fishers—particularly migrant workers—are at high risk of experiencing serious human rights violations while working on fishing boats and are disproportionately more likely to become victims. Migrant workers, specifically, are at risk of coercion and pressure by employment brokers and employment agencies to work on ships under the threat of force or through debt bondage [19]. Recent examinations into the fishing industry, including overfishing, unauthorized fishing, and a change in the recruitment of workers from high-and middle-income to lower-income countries, have led to an increase in the hiring of inexpensive migrant labor in the fisheries sector. These fishermen are especially at risk of being subjected to forced labor and human trafficking due to their insufficient training, limited language proficiency, and a lack of enforcement of safety and labor regulations [19].

### Costa Rican fishing industry

Costa Rica’s fishing sector represents a suitable case to reflect this pervasive issue. Many people from various countries migrate to Costa Rica due to poverty and political instability, which makes them vulnerable to exploitation. Those who move to Costa Rica out of desperation in search of employment and have an irregular migratory status are often more likely to accept unfavorable working conditions, as they have limited options in their home countries. Migrants face difficulty finding formal employment opportunities as they require a work permit and compliance with various regulations [20]. Formalized employment requires applying for a work permit, complying with social security, and paying taxes, meaning these barriers may result in pursuing work through unregulated channels [21,22]. The Costa Rican fishing industry mainly consists of artisanal fishing on the Pacific Coast, with a diverse range of boats from motorless vessels to advanced ships that can sail far off the coast [23,24]. According to a report by Arraya (2006), there are around 50 fishing cooperatives operating along the Central Pacific coast, but only 37 of them are registered with the government. This makes it difficult to gather accurate demographic statistics on the fishing industry workforce. However, it is clear that the fishing industry is a prominent sector for Costa Rica’s labor force and economy [25]. Fishing in Costa Rica is mainly carried out by decentralized artisanal fisheries, which are primarily located in the states of Limón and Puntarenas. These fisheries are not consistently registered with the government, and there is no fishing census, so it is difficult to determine the number of people employed in the fishing sector and their economic condition. Additionally, there is a lack of information on the role of women in the small-scale artisanal fishing sector, which makes it challenging to provide a detailed analysis of all the roles they play in this industry. This information gap hindered the ability to accurately estimate the entire population involved in Costa Rica’s fishing industry [20].

### Measuring exploitative labor

Conducting research on labor exploitation and trafficking is often challenging due to the unconventional sampling methods it requires. The reason for this is the “invisibility” of human trafficking, which owes in part to the demographics of the population most at risk. As discussed, obtaining accurate estimations regarding the prevalence of labor exploitation is a challenging task due to myriad reasons, such as the concealed and intricate nature of forced labor and exploitation, and inconsistent definitions and indicators have led to differences in prevalence estimations and interpretations. In considering these reasons, human trafficking researchers argue that there are two primary issues in gathering data that accurately reflect the scope and nature of labor trafficking and exploitation: definitional issues and methodological limitations [13,26].

Concerning definitional issues, De Cock (2007) highlights the challenges and significance of creating consistent trafficking measures across the field [27]. Despite the legal definition of human trafficking being widely agreed upon, various stakeholders such as judicial agencies, police agencies, and social service providers tend to employ criteria that align with their specific operational goals. Zhang (2022) argues that it can be challenging to convert the legal principles of the US Trafficking Victims Protection Act or Palermo Protocol into practical measures or questions that researchers can employ to gather field data. The research community has long struggled with defining human trafficking, especially when trying to gauge its prevalence. The main issue at hand is determining what should be considered as human trafficking violations versus exploitation versus workplace victimization, which are all often interdependent and overlap in various contexts.

There is no standardized method for estimating the prevalence of human trafficking and exploitation [28]; however, there is a growing consensus around certain measures and rules to count these activities [8]. Researchers have come up with various data collection strategies to estimate the prevalence of hard-to-reach populations in different contexts, each claiming superiority over the other methodologies [13,29]. There are various methods used to estimate the prevalence of the problem, such as probability-based sampling, mark-and-recapture methods, network scale-up methods, respondent-driven sampling, and link-tracing strategies. These varied methods can produce different estimates of the problem’s scale, which has resulted in different figures reported about the size and scale of the problem across the globe. The variation across international estimates has prompted some to question the validity of statements regarding the magnitude of the issue. Additionally, the relatively low number of arrests, charges, and convictions related to human trafficking has further raised doubts about the scale of the problem.

Researchers recognize that evaluation of complicated social and behavioral activities, that is, human trafficking and exploitation, is often influenced by specific cultural settings. Despite this, there are shared elements that enable us to identify some basic markers that we can employ to gather measurable data that can be compared. For example, the development of the Prevalence Reduction Innovation Forum (PRIF) common indicators can standardize measurement because it provides a set of common measures and counting rules based on the legal definition of human trafficking related to three elements: force, fraud, and coercion [30]. For the fishing industry specifically, there have been some advancements to estimate the risk of forced labor through the development of the Seafood Social Risk Tool (SSRT) by Monterey Bay Aquarium’s Seafood Watch and the Sustainable Fisheries Partnership. The tool uses a collection of country-, industry-, fishery-, and processing-level indicators to evaluate the likelihood of forced labor, human trafficking, and risky child labor in certain fisheries [31]. Other researchers have utilized metrics for environmental or resource governance performance [32], data from various companies, human rights organizations, and supply chain mapping to understand the nature and scope of labor trafficking and exploitation in the fishing industry [33].

While there are increased calls to enhance the methods to produce accurate estimates, emblematic of the January 2020 Executive Order Combating Human Trafficking and Online Child Exploitation in the United States [34], we seek to contribute to this call by exploring how the use of multiple data collection methods may supplement each other to produce a more robust picture of the issue. We know that instances of labor exploitation or trafficking are complex experiences defined by how broad and varied someone’s experience of harm is. Many factors contribute to these experiences, and they are largely context-specific. Truly understanding the scope of these issues is not viewing them through a lens that only counts how many types of harm a person has experienced, but instead explores how these complex instances of harm converge and manifest in varying degrees of exploitation. Therefore, we posit that the data collection method used to gather information on the experiences of exploited or trafficked workers will impact how we understand the degree of their exploitative experience in a given context.

## Materials and methods

### Data

Data for the current study come from a project aiming to understand the prevalence of forced labor in the Costa Rican fishing industry, which was conducted from 2021 to early 2023 [21]. Data were originally accessed for research purposes on January 10th, 2023. The large-scale study was developed out of the Prevalence Reduction Innovation Forum (PRIF), an initiative to standardize core indicators derived from the TVPA and Palermo Protocol in conjunction with the three primary elements of human trafficking (i.e., acts, means, and purpose). The study aimed to better understand how disparate methods of data collection differentially uncover the prevalence of exploitative labor [21]. These data were constructed via survey responses, which inquired about a range of personal and occupational characteristics, including 1) demographic characteristics; 2) debt and migration (e.g., recruitment or financial borrowing); 3) occupational characteristics (e.g., job type and work setting); and 5) exploitative labor conditions.

The population of the original study consisted of individuals who had been involved with the fishing industry in Costa Rica to varying degrees in the state of Puntarenas. Roles within the fishing industry could vary from crew, captain, dock helpers, pawn, etc., and the scale of work greatly varied from small-scale artisanal work to more large-scale industrial fishing (e.g., international tuna fishing). Surveys were administered to households during daylight hours, which, for the purpose of the original study, was defined to exclude tents, makeshift abodes, and boats. The original project was approved by the New York University: IRB. Protocol number: IRB-FY2022–5926. Beginning the surveys, participants were presented with and walked through consent forms. After reading through the consent form, participants gave verbal consent to participate in the study. Participants must have been at least 18 years old and were provided with contact information of project personnel and given IRB protocol numbers before verbally consenting. Consent responses were recorded in the survey instrument.

### Sampling

As previously mentioned, the current study uses data collected via two sampling methods in order to compare how broad categories of exploitation may change depending on sampling technique, as each brings their own tradeoffs that may make them inaccessible to certain researchers conducting work on exploitative labor. More detailed information can be found in Estimating the Prevalence of Forced Labor in the Fishing Industry in Costa Rica [21]. As the aim of the initial study was to uncover how methods of data collection affect labor trafficking and exploitation prevalence estimates, the current paper takes this initial goal further by investigating how the method of data collection affects the general understanding of how exploitative labor is practically experienced. Below, we provide an overview of how data collection methods differed among each sub-sample.

*PPS:* The first sub-sample in the current study is those that were sampled via a Probability Proportionate to Size (PPS) sample. Being a probability sample, studies of labor exploitation and trafficking should theoretically produce a relatively generalizable sample of exploitation amongst the specific working population of focus. However, probability samples are not always effective at measuring hidden communities, such as those who have experienced exploitative labor [35]. Using information from the 2011 Costa Rican census, researchers identified a number of coastal districts with more active fishing communities. A total of thirteen communities were sampled without replacement using Sampford’s PPS procedure [36]. In total, 75–80 surveys were conducted per community, as it was unclear whether the fishing population adequately scaled to the population of the community as a whole. A more detailed overview of the calculations made to determine precise sample sizing can be found in [21]. Practically, researchers approached households within said communities and had one person–the adult with the birthday closest to the date of data collection–take the survey from each household.

*VLTS:* The second sub-sample of respondents was collected through a Vincent Link-Tracing Sampling (VLTS) technique. Unlike the probability sampling method outlined above, VLTS is a network-based strategy similar to Response-Driven Sampling (RDS)--a variation of traditional snowball sampling. Network-based sampling methods present their own benefits, such as their ability to access hidden populations [37], but have drawbacks in terms of bias and generalizability [35]. These variations of snowball sampling aim to address some of its major limitations, notably the lack of generalizability stemming from the unequal selection probabilities endemic to respondent recruitment [38]. Here, the research team further aimed to address these limitations by increasing the initial sample size from which respondent recruitment began. Initial participants were identified and given remuneration for their participation, and were then given the opportunity to recruit three new participants for further remuneration. The base idea of this method was to still maintain the benefits of snowball sampling (i.e., the ability to find hidden populations), while reducing bias that affects generalizability [39].

Ideally, this variation on snowball sampling limits the size of any individual network while diversifying the initial wave of study participants. While this method may not deeply interact with a given community, its breadth ultimately produces less biased results. As with the PPS sub-sample, more intricate details on data collection for the VLTS sample can be found in [21].

For the current paper, anyone who had not responded to at least half of the included labor exploitation items (outlined in more detail below) was dropped from the survey, giving a starting overall sample size of 823 for the PPS sample and 946 for the VLTS sample.

### Measurements

#### Latent class items.

We employed a Latent Class Analysis (LCA) model to explore how labor exploitation may be experienced by the respondents depending on the type of sampling method used. The LCA model is a statistical technique for detecting unidentified group affiliation among participants by analyzing either or both categorical and continuous observed variables. We constructed the model using dichotomous items measuring labor exploitation (1 = No experience of specific exploitation, 2 = Experience of specific exploitation). Most of the exploitation-based items were developed within the broader context of PRIF, meaning these items have been used in a variety of labor exploitation prevalence estimates studies [30]. Information regarding the construction of PRIF indicators, as well as information of validation, can be found in [10]. Conceptually, PRIF indicators measure five conceptual domains of exploitative labor, including 1) recruitment, 2) employment, practices, and penalties, 3) personal life and properties, 4) freedom of movement, and 5) violence and threats of violence.

In total, the current study includes 38 PRIF items and six non-PRIF items measuring other components of labor exploitation, which include having savings confiscated, being belittled in front of peers, being ostracized from peers, being forced to work when one refused to, not being allowed visitors, and not being able to seek medical services. Only one item: “Made to be available day and night without adequate compensation outside the scope of the contract” had to be removed from analyses due to missing data issues. The missing data were due to the fact that most fishers while out at sea are expected to work around the clock. A full breakdown of the exploitation items and their frequencies can be found in Table 1.

**Table 1:** LCA item descriptives.

Variable	PPS		LTS		Overall	
N	(%)	N	(%)	N	(%)
Charged fees	102	14.8	96	15.4	198	15.1
Reduced value of goods produced	189	27.4	218	34.9	407	31.0
Reduced compensation through fees	81	11.8	63	10.1	144	11.0
Controlled through blackmail	13	1.9	11	1.8	24	1.8
Controlled through religious retribution	6	0.9	2	0.3	8	0.6
Controlled through blacklisting	39	5.7	45	7.2	84	6.4
Controlled through family isolation	7	1.0	10	1.6	17	1.3
Controlled through friend isolation	10	1.5	11	1.8	21	1.6
Controlled through sex acts	5	0.7	2	0.3	7	0.5
Taken identity papers	11	1.6	8	1.3	19	1.4
Forbidden from leaving worksite	41	6.0	29	4.6	70	5.3
Kept under surveillance	53	7.7	32	5.1	85	6.5
Kept in isolated place	16	2.3	2	0.3	18	1.4
Locked in workplace	11	1.6	6	1.0	17	1.3
Non-work hours travel restriction	37	5.4	39	6.2	76	5.8
Phone confiscated	18	2.6	6	1.0	24	1.8
Family communication restriction	20	2.9	11	1.8	31	2.4
Coworker communication restriction	21	3.0	12	1.9	33	2.5
Other communication restriction	18	2 6	15	2.4	33	2.5
Pushed or shook	31	4 5	45	7.2	76	5.8
Slapped or twisted arm	6	0.9	16	2.6	22	1.7
Punched with fist	15	2.2	22	3.5	37	2.8
Kicked or dragged	3	4	9	1.4	12	0.9
Attempted strangulation or burning	2	0.3	5	0.8	7	0.5
Attacked with weapon	14	2.0	20	3.2	34	2.6
Forced to do unwanted sex act	8	1.2	3	0.5	11	0.8
Forced to be photographed	6	0.9	5	0.8	11	0.8
Used sexual violence against loved one	6	0.9	4	0.6	10	0.8
Smashed things to intimidate	30	4.4	35	5.6	65	5.0
Threatened physical violence	27	3.9	48	7.7	75	5.7
Threatened physical violence against loved one	14	2.0	24	3.8	38	2.9
Used physical violence against loved one	11	1.6	27	4.3	38	2.9
Imposed debt	54	7.8	63	10.1	117	8.9
Could not seek medical services	36	5.2	40	6.4	76	5.8
Not allowed visitors	17	2.5	16	2.6	33	2.5
Forced to work when refused to	26	3.8	26	4.2	52	4.0
Confiscated savings	9	1.3	7	1.1	16	1.2
Belittled in front of peers	51	7.4	68	10.9	119	9.1
Ostracized from peers	8	1.2	10	1.6	18	1.4
Felt obliged during recruitment	30	4.4	32	5.1	62	4.7
Lied about nature of work	34	4.9	44	7.1	78	5.9
Do things differently than told	22	3.2	34	5.4	56	4.3
Withheld compensation	39	5.7	38	6.1	77	5.9
Lose compensation if quit	46	6.7	32	5.1	78	5.9

#### Control items.

Outside of labor exploitation items, we include a number of personal and occupational measures in the final analytic step of the current paper. Full descriptive statistics for both sub-samples and the overall sample are found in Table 2. Regarding personal demographic information, we additionally include information for the district in which individuals were sampled (dummy coded 0/1), gender (1 = female, 0 = non-female), education (ordered variable for education level), and monthly expenses (ordered variable for expense ranges). The final piece of personal demographic data we measure is whether an individual receives Costa Rican Mixed Institute of Social Assistance (IMAS) benefits (coded as 1 = yes, 0 = no).

**Table 2:** Descriptive statistics.

Variable	PPS: 823 N (%)	LTS: 946 N (%)	Overall: 1769 N (%)
Gender			
Female	366 (44.47%)	281 (29.7%)	647 (36.57%)
Male	451 (54.8%)	657 (69.45%)	1108 (62.63%)
Non-binary	5 (0.61%)	4 (0.42%)	9 (0.51%)
Children			
Children	684 (83.11%)	789 (83.4%)	1473 (83.27%)
Education			
Cannot read or write/Illiterate	34 (4.13%)	35 (3.7%)	69 (3.9%)
No Formal Education/Literate	130 (15.8%)	192 (20.3%)	322 (18.2%)
Primary School	462 (56.14%)	547 (57.82%)	1009 (57.04%)
Secondary School	73 (8.87%)	61 (6.45%)	134 (7.57%)
High School Degree	104 (12.64%)	89 (9.41%)	193 (10.91%)
Vocational School/some College	6 (0.73%)	9 (0.95%)	15 (0.85%)
Bachelor’s Degree	3 (0.36%)	13 (1.37%)	16 (0.9%)
Monthly expenses			
Prefer not to say/Don’t know	3 (0.36%)	7 (0.74%)	10 (0.57%)
Less than 100,000	768 (93.32%)	845 (89.32%)	1613 (91.18%)
Between 100,001–300,000	49 (5.95%)	79 (8.35%)	128 (7.24%)
Between 300,001–500,000	3 (0.36%)	7 (0.74%)	7 (0.4%)
Between 500,001–700,000	0 (0%)	3 (0.32%)	3 (0.17%)
More than 700,001	0 (0%)	5 (0.53%)	8 (0.45%)
Livelihood			
IMAS benefits	179 (21.75%)	207 (21.88%)	386 (21.82%)
INCOPESCA license	133 (16.16%)	290 (30.66%)	423 (23.91%)
Relationship Status			
Currently married	367 (44.59%)	547 (57.82%)	914 (51.67%)
Divorced	25 (3.04%)	49 (5.18%)	74 (4.18%)
Never married	379 (46.05%)	306 (32.35%)	685 (38.72%)
Separated	41 (4.98%)	27 (2.85%)	68 (3.84%)
Widowed	10 (1.22%)	17 (1.8%)	27 (1.53%)
District			
Chacarita	132 (16.04%)	86 (9.09%)	218 (12.32%)
Chira	96 (11.66%)	134 (14.16%)	230 (13%)
Chomes	77 (9.36%)	132 (13.95%)	209 (11.81%)
El Roble	67 (8.14%)	64 (6.77%)	131 (7.41%)
Lepanto	67 (8.14%)	72 (7.61%)	139 (7.86%)
Manzanillo	136 (16.52%)	113 (11.95%)	249 (14.08%)
Puntarenas	63 (7.65%)	133 (14.06%)	196 (11.08%)
Tárcoles	0 (0%)	72 (7.61%)	72 (4.07%)
Recruitment			
I do not know	3 (0.36%)	5 (0.53%)	8 (0.45%)
No	348 (42.28%)	492 (52.01%)	840 (47.48%)
There was no fee	458 (55.65%)	413 (43.66%)	871 (49.24%)
Yes	14 (1.7%)	32 (3.38%)	46 (2.6%)
Subcontract			
I do not know	2 (0.24%)	2 (0.21%)	4 (0.23%)
No	426 (51.76%)	618 (65.33%)	1044 (59.02%)
Yes	340 (41.31%)	281 (29.7%)	621 (35.1%)

Outside of personal demographic information, we control for a myriad of occupational characteristics. First, we control for whether a respondent had an INCOPESCA license (Instituto Costarricense de Pesca y Acuicultura), which is a requirement for artisanal or industrial fishing in Costa Rica (coded 1 = has license, 0 = does not have/does not know). Next, our multivariate analyses account for both the role individuals have within their job and the type of fishing that defines them. For role, we include four dichotomous variables (coded 1 = has role, 0 = does not have role), specifically measuring whether respondents were 1) boat owner, 2) crew, 3) pawn, and 4) shrimp peeler. Regarding the broad type of fishing (coded 1 = worked in said industry, 0 = not), we control for whether individuals worked in either small-scale artisanal fishing or medium-scale artisanal fishing, with industrial fishing being left out as a reference.

Finally, we include variables to account for whether a respondent had to pay a recruitment fee for their job or if an individual worked for a subcontractor. Below, we include information on whether the respondent either had a recruitment fee paid for by their employer (coded 1/0) or did not have any recruitment fee (coded 1/0) with not-paying a recruitment fee as a reference. Similarly, we also include whether the respondent worked for a sub-contractor, or someone who administers work tasks who is not a direct employer (1 = no, 0 = yes). Finally, one of our analytical steps includes a variable for a sub-sample, titled “data source.” This variable is a dichotomous measure for whether an individual comes from the PPS sample or the VLTS sample (coded 1 = pps, 0 = vlts).

### Analytic strategy

The current analytic process takes place in two stages: 1) LCA on exploitative labor items and 2) Regression on found latent classes. We specifically run each analytic stage on each sub-sample and the overall sample to garner a more complete picture of latent forms of exploitation and their alterations based on data collection methodology. LCA serves as a tool that finds mutually exclusive latent constructs underlying study data [40]. By using methodologies that identify specified groups, it is additionally possible to understand what study variables distinguish the varying latent classes. Due to the non-randomness of missing data, we elect to run our analyses using complete case analysis, bringing the analytic sample size to 1313 (689 PPS sample, 624 VLTS sample). In order to determine whether data were missing at random, a dichotomous variable was created and correlated with all study variables. The missing variable had a significant correlation with a small number of exploitation and district items. In order to better understand how missingness of data is associated with study variables, S1 Table in the supplementary materials provides an overview of key characteristics for those that were removed for complete case analysis and those included in the LCA and subsequent analyses. The rightmost column indicates any significant differences via chi-square tests between the two sub-samples.

First, we ran a series of LCA models with disparate class specifications on each of the three groups of respondents. Our choice of best-fitting model was ultimately determined by the lowest Bayesian Information Criterion (BIC) value [40]. After finding the best-fitting models within each sample, both entropy values and average latent class probabilities act as diagnostic tools to better evaluate the found classes. Entropy is a metric that evaluates the separation between classes, ranging from 0 to 1 [41]. Generally, entropy values above.8 are considered acceptable [40]. Average latent class probabilities, much like entropy, act as a diagnostic tool that ranges from 0 to 1, with values over.9 considered ideal [42]. In essence, we use LCA to find underlying latent categories of exploitative labor amongst all three samples. Table 3 conveys the fit statistics and entropy values for each sample with classes ranging from 1 to 5. For the overall sample, a 3-class model was determined to have the best fit, and a 2-class model best fit both of the sub-samples individually. While model diagnostics such as entropy and average posterior probability remained acceptable for 3-class solutions for both sub-samples, the model fit statistics were best on average for the 2-class retention. Ultimately, with BIC being lowest and entropy being highest for both 2-class solutions, we elected to retain two classes for the sub-samples.

**Table 3:** Model comparison table.

Model Log-likelihood		DF	BIC	aBIC	cAIC	Likelihood-ratio	Entropy
*Overall Sample*							
Model 1	−9206.263	1269	18728 449	18588.682	18772.449	9153.439	*–*
Model 2	−7779.558	1224	16198 142	15915.431	16287.142	6300.028	0.889
Model 3	−7535.051	1179	16032 231	15606.575	16166.231	5811.014	0.784
Model 4	−7426.740	1134	16138 712	15570.113	16317.712	5594.392	0.777
Model 5	−7316.634	1089	16241 604	15530.061	16465.604	5374.180	0.753
*PPS Sample*							
Model 1	−4618.025	645	9523 600	9383.893	9567.600	4847.229	–
Model 2	−3829.382	600	8240 400	7957.812	8329.400	3269.944	0.908
Model 3	−3692.113	555	8259 948	7834.477	8393.948	2995.405	0.829
Model 4	−3609.223	510	8388 254	7819.901	8567.254	2829.625	0.608
Model 5	−3536.974	465	8537 843	7826.608	8761.843	2685.128	0.589
*LTS Sample*							
Model 1	−4536.783	580	9356 757	9217.063	9400.757	4769.095	–
Model 2	−3859.701	535	8292 219	8009.657	8381.219	3414.930	0.875
Model 3	−3720.574	490	8303 593	7878.163	8437.593	3136.677	0.813
Model 4	−3654.680	445	8461 432	7893.133	8640.432	3004.889	0.805
Model 5	−3592.656	400	8627 009	7915.842	8851.009	2880.840	0.769

Upon finding the best-fitting modes for each sample, a predicted class variable was constructed, categorizing each survey respondent into one of the latent classes. We then ran a series of regression analyses to determine how personal and occupational characteristics altered the odds an individual would fall within each latent class. For the overall sample, an ordinal logistic regression was run on the three-class solution. The Brant test was used to determine whether the predicted class outcome met the assumption of parallel lines, finding that the assumption held. For both of the sub-sample, logistic regression was performed with the first class coded 0 and the second class coded 1. We conducted our analyses in R, using the packages poLCA to construct our LCA models [43], and pscl for ordinal logistic regression models [44].

## Results

### By sample results

**Fig 1:**
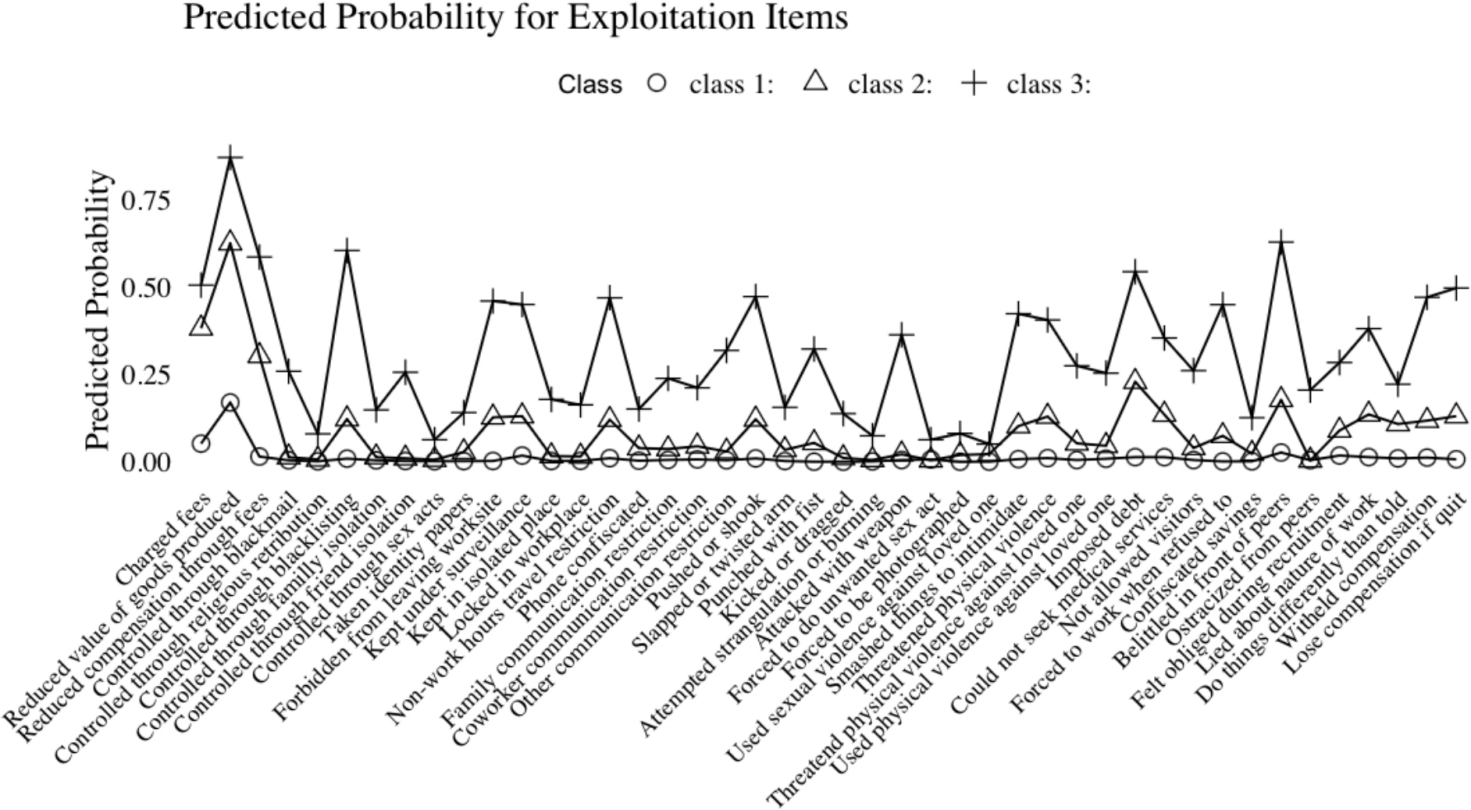
Posterior probabilities of exploitation items for overall LCA.

Results of the overall sample’s LCA are represented by item probabilities in Fig 1. Within each sample, the results of our LCA present latent forms of exploitative labor defined by the degree of exploitation rather than necessarily variations in the types of exploitation experienced.

For the entire sample, fit statistics indicate a 3-class solution, with average posterior probabilities ranging from.91 to.97. Similarly, the three-class model has an entropy value of.784. Within this group, all three latent classes were defined by a diversity of exploitative conditions but differentiated by a dampening of the probabilities of having a given experience. Less diverse and likely exploitative experiences define class one (n = 974). For this group of individuals, the most likely forms of exploitation are a reduction in the value of goods produced or being belittled by one’s peers. The second latent class (*n* = 279) was defined by a greater variety of exploitation, predominantly seeing wage-related issues (e.g., having the value of goods produced devalued, being charged fees, or having reduced compensation through fees). Finally, the third latent class (*n* = 60) was the most highly exploited class, experiencing a wide range of adverse labor and seeing a large increase in what may be considered “classic” (e.g., severe) trafficking indicators (e.g., restrictions of movement or communication).

For each of the two sub-samples, fit statistics designate a two-class solution. For the VLTS sample (*n* = 624), average posterior probabilities ranged from.94 to.98 and an entropy value of.875. The first latent class (*n* = 526), was similar to the first latent class in the overall sample. Specifically, this class was defined by lower probabilities of exploitation, with the most prominent individual experiences having reduced values of goods produced and being charged fees. The second latent class (*n* = 98) was defined by a much greater diversity of exploitative experiences, with items (aside from having the value of good reduced) such as threats of physical violence or restriction of communicative freedom predominantly defining this class. While entropy values remain acceptable for 3-class solutions, the more holistic suite of diagnostics for both fit (particularly BIC) and post-hoc metrics (e.g., average posterior probability, class membersip, and entropy) were strongest amongst the 2-class solution.

Turning toward the PPS sample (*n* = 689), average posterior probabilities ranged from.94 to.99 with an entropy of.908. Much like the VLTS sample, the first latent class (*n* = 603) was the more minimally exploited group, being more defined by the same forms of exploitation as those in the PPS sample. The second latent class (*n* = 86) appeared to exhibit higher degrees of exploitation-like experiences more closely aligned with restrictions of physical freedom (e.g., being forbidden from leaving the workplace or having identity papers taken) and issues of lost compensation (e.g., withheld compensation or losing compensation if they quit).

### Comparative results

**Fig 2:**
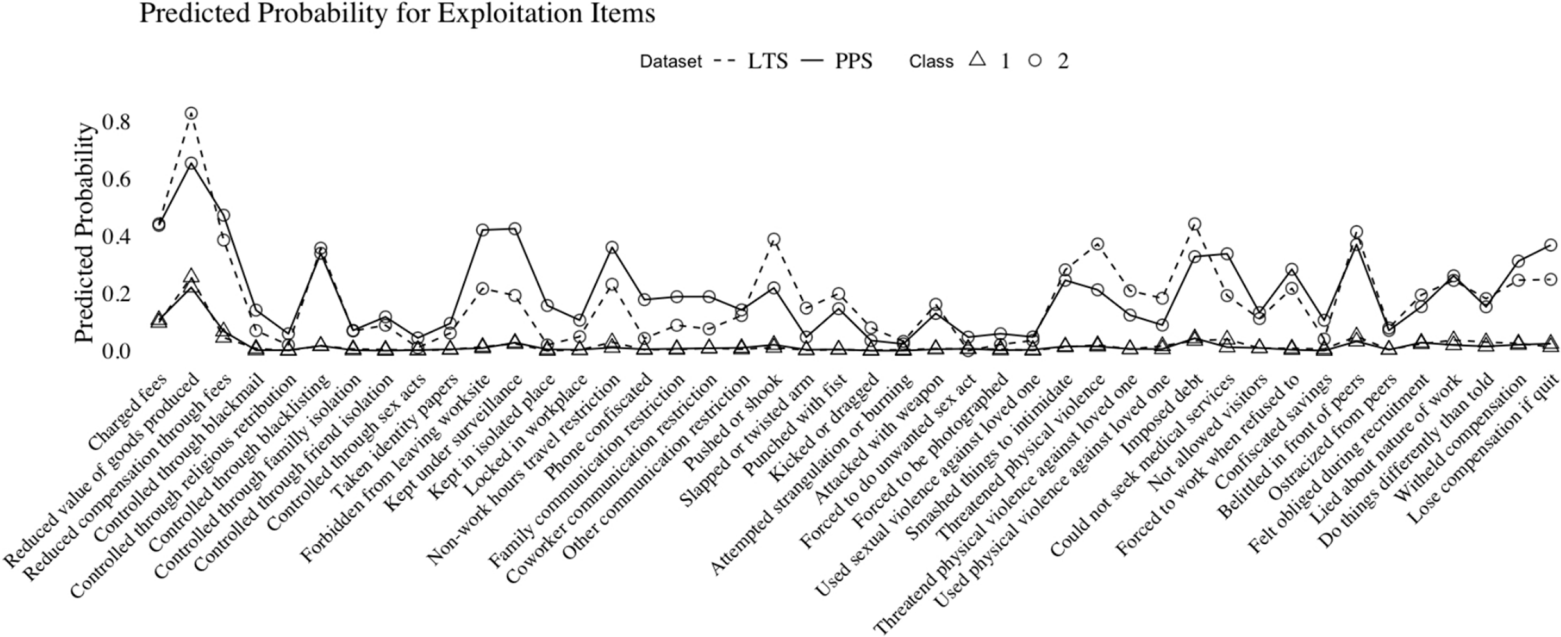
Posterior probabilities of exploitation items for both subsample LCA models.

Fig 2 presents item probabilities by each dataset, and each class is overlaid for better visual comparisons. For the individuals in either sample who fall into the first latent class or the more minimally exploited class, experiences did not differ nearly at all. Almost all of these study participants across both PPS and VLTS samples experienced exploitation defined by devaluing of goods produced, unnecessary fees, and belittling by one’s peers.

Much like the first class, both second classes, or the more highly exploited workers, had a generally similar structure to their exploitation, though it is here where differences become more apparent. Those in the VLTS sample had their second latent class more clearly defined by communication restriction, physical violence, or threatened violence. Conversely, those in the PPS sample had the more highly exploited class marked by physical movement restriction or wage violations.

Comparing the results side by side, it is clear that more heightened forms of exploitation have both probabilistic and qualitative differences depending on the method of data collection. Those in the PPS sample had a highly exploited class that aligns with more “classic” measures of labor trafficking, while those in the VLTS sample saw latent forms of exploitation that more closely mirror conceptualizations of violent workplaces as opposed to trafficking-like workplaces.

### Regression analysis results

The final step in our analysis involved transforming their latent classes into predicted-class outcome variables and determining which study measures contribute to respondents being in a given latent class, as presented in Table 4. Looking first at the overall sample, we initially see a series of districts decreasing the odds of moving up a latent class. Given that each latent class increases in diversity and the probability of experiencing a wide range of latent classes, we may interpret this as certain areas decreasing the odds of being more intensely exploited. Similarly, women and those not working under a sub-contract had a decrease in the odds of being in the class characterized by more complex exploitation. Only one variable–having a recruitment fee that was paid for by their employer–significantly increased the odds of being in the more highly exploited class. Finally, the data source had a significant association with the predicted class outcome, as those in the VLTS sample were more likely to be in the more highly exploited classes.

**Table 4:** Overall sample ordinal logistic regression of latent classes.

Variables	Odds Ratio (95% CI)
Chacarita	0.86 (0.51, 1.47)
Chira	0.38 (0.19, 0.75)**
Chomes	0.25 (0.14, 0.46)***
El Roble	0.59 (0.32, 1.11)
Lepanto	0.34 (0.17, 0.68)**
Manzanillo	0.2 (0.11, 0.37)***
Puntarenas	0.79 (0.5, 1.26)
Ta´rcoles	0.84 (0.39, 1.82)
Female	0.58 (0.4, 0.86)**
Education	0.95 (0.83, 1.08)
Monthly Expenses	1.21 (0.9, 1.63)
Small-scale artisanal fishing (panga)	1.23 (0.88, 1.73)
Medium artisanal fishing (boat, longline)	1.62 (0.97, 2.71)
Boat Owner	1.22 (0.58, 2.59)
Crew	0.84 (0.53, 1.34)
Pawn	0.82 (0.56, 1.19)
Shrimp Peeler	0.59 (0.32, 1.07)
IMAS Benefits	1.23 (0.86, 1.74)
INCOPESCA License	1.22 (0.87, 1.69)
No recruitment fee	0.98 (0.72, 1.34)
Recruitment fee was paid	3.32 (1.51, 7.29)**
No Subcontract	0.58 (0.44, 0.76)***
Data Source	0.6 (0.44, 0.8)***
Ta´rcoles	0.45 (0.21, 1)
1|2	1 (0.51, 1.93)
2|3	8.8 (4.39, 17.64)***
Pseudo R-squared	0.1
Number of Observations	1313

* p < 0.05, ** p < 0.01, *** p < 0.001.

Individually, the pattern of results remains nearly identical for those in the VLTS sample–aligning with the data source results from the overall regression analysis. Interestingly, within those in the PPS sample, working in medium-sized artisanal fishing significantly increased the odds of being in the more exploited class. Overall, these results further convey that there are some small differences in how the data collection method alters the measurement of exploitative labor. However, what seems to greatly influence whether a worker will experience more or less exploitation is defined by the type of work they are in and the location in which they are working. All regression results for the two sub-samples are displayed in Table 5.

**Table 5:** Logistic regression for each sample latent class outcome.

Variables	PPS Odds Ratio (95% CI)	LTS Odds Ratio (95% CI)
Boat Owner	1.17 (0.3, 4.49)	1.31 (0.22, 7.75)
Chacarita	1.03 (0.37, 2.83)	0.69 (0.26, 1.81)
Chira	1.23 (0.39, 3.88)	0.16 (0.04, 0.66)*
Chomes	0.57 (0.19, 1.71)	0.1 (0.03, 0.38)***
Crew	1.14 (0.52, 2.52)	0.58 (0.25, 1.31)
Education	1.11 (0.87, 1.42)	0.99 (0.78, 1.26)
El Roble	0.85 (0.29, 2.53)	0.24 (0.06, 0.96)*
Female	0.69 (0.33, 1.42)	0.83 (0.38, 1.78)
IMAS Benefits	1.78 (0.91, 3.48)	0.86 (0.43, 1.73)
INCOPESCA License	0.82 (0.45, 1.5)	1.72 (0.9, 3.3)
Lepanto	0.15 (0.02, 1.27)	0.08 (0.01, 0.43)**
Manzanillo	0.26 (0.07, 1.02)	0.17 (0.06, 0.52)**
Medium artisanal fishing	4.61 (1.82, 11.71)**	0.95 (0.39, 2.34)
Monthly Expenses	1.45 (0.96, 2.2)	1.81 (0.81, 4.04)
No recruitment fee	1.38 (0.76, 2.52)	1 (0.54, 1.85)
No Subcontract	0.3 (0.18, 0.49)***	0.45 (0.27, 0.76)**
Pawn	0.67 (0.3, 1.49)	0.59 (0.31, 1.13)
Puntarenas	0.72 (0.3, 1.76)	0.76 (0.32, 1.79)
Recruitment fee was paid	4.6 (1.35, 15.62)*	7.95 (1.56, 40.49)*
Shrimp Peeler	1.15 (0.31, 4.33)	0.43 (0.15, 1.21)
Small-scale artisanal fishing	1.52 (0.83, 2.76)	1.07 (0.54, 2.13)
Ta´rcoles	1.48 (0.5, 4.39)	0.58 (0.22, 1.53)
Intercept	0.11 (0.03, 0.36)***	0.43 (0.11, 1.66)
Number of Observations	689	624
Pseudo R-squared	0.14	0.17

* p < 0.05, ** p < 0.01, *** p < 0.001.

## Discussion

The current paper continues previous work that has made significant strides in unpacking what it means to experience labor exploitation [7,13,45,46]. The current study finds a range of two to three latent classes, which ultimately point toward varying degrees by which people experience exploitation. The lower-level latent classes for the overall sample and each sub-sample contained the highest number of participants and were characterized by less diverse and probable instances of exploitation. Additionally, regression analyses, which are used to unpack personal and occupational characteristics associated with the disparate latent classes, convey how the district in which data were collected and the type of fishing is most associated with experiencing higher degrees of exploitation.

Results of the LCA and regression analyses convey several interesting findings and theoretical implications for future labor-based research. First, LCA–particularly in the context of victimization [47]--often finds classes defined by different individual forms of victimizing experiences. Perhaps the unique LCA results here–emphasizing more degree or complexity of exploitation than thematic type of exploitation–are contingent on the homogeneity of the unique context in which these specific experiences are felt, that is, the workplace. The current study solely examines individuals involved in the fishing industry in Costa Rica, meaning that exploitative experiences may not vary in type but more in magnitude as broader contextual factors remain constant.

There is a growing contingent of literature on the contextual and individual causes of negative working environments [14]; however, this work still has notable gaps, particularly when unpacking how environmental and occupational context affects the conditions of one’s labor. Some growing evidence shows that highly specified characteristics of one’s unique occupational landscape–e.g., hours worked, location of the job, physical tasks– may be relatively predictive of exploitative experiences [45]. Prior data collection efforts reveal that disparate occupational contexts have a tendency to produce distinct forms of exploitative labor [7]. Given the relatively homogeneous work landscape examined in this paper, the findings build on prior research to suggest that the forms of exploitative labor experienced by a given worker are largely shaped by the exploitative practices available within that context. Available exploitation is going to be largely defined by work and where the work is physically located, which we find in the results of our regression analyses when the district of the respondent and occupational characteristics are the primary delineators of latent classes. Our findings here indicate that the structure of exploitation likely depends more on how different types of work require different tasks, physical needs, and how the industry operates in different environments. That is, an individual working in an industry with high gender parity and laborious tasks will experience different forms of unsafe labor than those with more heterogeneous working populations in an office setting.

The LCA results present some interesting findings regarding the method by which data is collected. Data collection methods have had some effect in altering prevalence estimates in the contexts of exploitative labor, but these results are not dramatic [21]. These findings align well with the results here as this previous work indicates that data collection methods lead to some variations in results–though not enough to dramatically alter the broader understanding of the issue. Here, both the PPS sample and VLTS sample found two distinct classes of exploitative labor with highly similar patterns–one less exploited class and one more greatly exploited class. Perhaps these results indicate the structure of exploitative labor is well captured through a broad range of data collection methods, particularly for a sample of individuals who are relatively homogeneous from an occupational/industry perspective. Results add credence to the idea that the two strands of research on exploitative and victimizing labor–that being prevalence estimates and underpinning mechanisms that cause exploitative labor–may be affected by data collection methods, but provide enough cohesion in their overall understanding of the issue that researchers should focus on data collection efforts that are going to be the most implementable for their specific work.

Given the novelty of the focus of the current paper, it is difficult to know exactly where the differences between the two methods originate. It is clear that while the method used to collect data may not hinder the overall consensus about the prevalence and experience of exploitation, it does create some clear distinctions that should unquestionably be centralized in future research. Perhaps in certain country and occupational contexts, more severe forms of exploitation have an isolating effect on individuals–thereby minimizing social networks and reducing the chances of finding these experiences through network-based sampling techniques. This, in part, may be due to the unique and esoteric nature of work and labor, which occupies a theoretically, philosophically, and practically distinct position in the modern social world [48].

It is also important to note that some findings, such as the fact that women are less likely to be in the more highly exploited classes, may be due to the nature of the industry of central focus here. Fishing being a male-dominated industry could cause a lower likelihood of reporting for women, or women may more often be in positions within the industry that are less exposed to the forms of exploitation measured in this paper.

Like all studies, the current research contains limitations that must be addressed. The first and most clear limitation regards the volume of missing data. It is unideal to have missing data to the degree presented in the current paper, though this is a noted problem in research that seeks to address highly sensitive and hard-to-reach populations [49]. Due to both computational constraints with widely available methods of conducting LCA and conceptual limitations with the non-random nature of some of the missing data, no method of approaching the issue is perfect. Packages like poLCA have no direct way of implementing strategies like multiple imputation nor full information maximum likelihood (FIML). To help understand the context of missing data, and its potential biases it interjects, we compared the sample excluded due to missingness with those included to get an idea of how the two samples differ. While there are a handful of clear differences between the two groups, these are primarily along geographic lines, and less on demographic information. This does mean results from a geographic stance warrant an extra level of caution in their interpretation. Despite this limitation, the sample size for the current paper remains relatively high and allows us to maintain confidence in the current findings.

An additional limitation that needs addressing is the rarity of some of the exploitation items in the study. Using events with low variance can produce biased findings and requires adequate prudence in LCA interpretation. While many of the exploitation items were rarely endorsed, it is important to note that this does reflect the challenging nature of uncovering and measuring sensitive topics like labor exploitation amongst hard-to-reach populations. Even with low variance, the LCA models all demonstrated good fit with entropy values and average posterior probabilities above the literature-supported cutoffs. The construction of well-fitting LCA models on rarely indicated exploitation measures further reflect the promising nature of this line of work for future studies in disparate contexts.

There were additional limitations outlined by the initial study collection report, and more details can be found in Dank, Zhang, Vincent, Stoeltje, et al. (2024). One of the central components of the overall study is to understand how the bias of methods like VLTS affect prevalence, which we extend in this paper, but it is important to note that those biases still exist in the data using the link tracing methods. Despite study limitations, the results present unique and useful insights into the idea of exploitative labor and reveal implications for theory, research, and practice.

Thinking through the implications the current study has for theory, it is clear that the context of labor and occupations has a fairly distinct effect on the negative experiences of workers and employees. Given this fact, it may be fruitful for future research on labor and occupations, specifically exploitative and victimization experiences in the context of labor and occupations, to be highly cognizant of this contextual and hierarchical nature. Generally, the literature on labor has a tendency to unpack the manner in which broad societal institutions affect the landscape of work–such as in the context of shifting towards digital on-demand work [50,51]–or focus on the more micro individual level experiences which impact negative labor outcomes. However, this study potentially points towards a theoretical complexity of labor in which individuals exist in a specific working context, which itself exists in a broader environmental context.

The current findings also present practical implications for future research studies seeking to better understand the dimensionality of exploitative labor and its underlying causal mechanisms. While attempts to estimate the prevalence of labor exploitation may be notably affected by methods of data collection [21], this may be less true for studies for researchers hoping to better understand how exploitative labor is practically experienced. This means that future scholars may have a wider set of data collection tools at their disposal to uncover the nature of labor exploitation without widely biasing their results. Perhaps ongoing work could examine how data collection approaches shape the underlying processes of exploitative labor through the use of hybrid data collection methods. Finally, the current research includes potential implications for policy and practice, as resources allotted to those who have experienced exploitative labor may need to tailor their recommendations based on the specific context (both environmental and occupational) of an individual’s working history.

## Conclusion

Results of the current study indicate that within the singular industry of fishing in Costa Rica, latent classes of exploitative labor may be based more on the magnitude of exploitation rather than the substantive type of exploitation. When further attempting to understand what distinguishes workers in the current sample from landing in the more highly exploited class, we find occupational and environmental characteristics, such as the type of fish or district in which a respondent works, being some of the primary distinguishing factors. Further, we find that the method of collecting data–whether it be probabilistic-based or a link tracing method, which has historically been used to sample hard-to-reach populations–does not have a tendency to alter the latent classes of exploitation. While there are some distinct differences in how highly exploited classes in the PPS sample appear as opposed to the VLTS sample, it is also clear that our broad understanding of exploitative labor would be fundamentally different using solely one data collection technique.

## Supporting information

S1 Table Comparison of study variables for the overall sample, the sample of those removed due to complete case analysis, and the final analytic sample. (DOCX)
