# Supplementary material for: Measuring and understanding labor exploitation on a sample of workers in the Costa Rican fishing industry
Source: PLoS One. 2026 Feb 6;21(2):e0341336. doi: 10.1371/journal.pone.0341336 (PMC12880695; doi:10.1371/journal.pone.0341336)
Supplement: S1 Table — (DOCX) [file pone.0341336.s001.docx]

**Supporting Information**

| *Table S1: Missingness Comparison* | | | | |
| --- | --- | --- | --- | --- |
|  | **Overall** | **Excluded** | **Excluded** | **p** |
| **n** | 1769 | 456 | 1313 |  |
| **Female: Mean (SD)** | 0.37 (0.48) | 0.35 (0.48) | 0.37 (0.48) | 0.380 |
| **Male: Mean (SD)** | 0.63 (0.48) | 0.64 (0.48) | 0.62 (0.49) | 0.407 |
| **Non-binary: Mean (SD)** | 0.01 (0.07) | 0.01 (0.08) | 0.00 (0.07) | 0.604 |
| **Has children: Mean (SD)** | 0.83 (0.37) | 0.85 (0.36) | 0.83 (0.38) | 0.359 |
| **Education: Mean (SD)** | 2.10 (1.03) | 2.08 (1.01) | 2.10 (1.04) | 0.719 |
| **Monthly Expenses: Mean (SD)** | 1.09 (0.37) | 1.05 (0.25) | 1.10 (0.40) | 0.021 |
| **IMAS Benefits: Mean (SD)** | 0.22 (0.41) | 0.25 (0.43) | 0.21 (0.41) | 0.056 |
| **INCOPESCA License: Mean (SD)** | 0.24 (0.43) | 0.29 (0.46) | 0.22 (0.41) | 0.001 |
| **Data Source (PPS): Mean (SD)** | 0.53 (0.50) | 0.56 (0.50) | 0.52 (0.50) | 0.152 |
| **Currently married: Mean (SD)** | 0.52 (0.50) | 0.58 (0.49) | 0.49 (0.50) | 0.001 |
| **Divorced: Mean (SD)** | 0.04 (0.20) | 0.02 (0.15) | 0.05 (0.22) | 0.014 |
| **Never married: Mean (SD)** | 0.39 (0.49) | 0.34 (0.47) | 0.40 (0.49) | 0.022 |
| **Separated: Mean (SD)** | 0.04 (0.19) | 0.04 (0.20) | 0.04 (0.19) | 0.678 |
| **Widowed: Mean (SD)** | 0.02 (0.12) | 0.01 (0.11) | 0.02 (0.13) | 0.671 |
| **Chacarita: Mean (SD)** | 0.12 (0.33) | 0.06 (0.23) | 0.15 (0.35) | <0.001 |
| **Chira: Mean (SD)** | 0.13 (0.34) | 0.34 (0.47) | 0.06 (0.23) | <0.001 |
| **Chomes: Mean (SD)** | 0.12 (0.32) | 0.14 (0.35) | 0.11 (0.31) | 0.088 |
| **El Roble: Mean (SD)** | 0.07 (0.26) | 0.02 (0.15) | 0.09 (0.29) | <0.001 |
| **Lepanto: Mean (SD)** | 0.08 (0.27) | 0.11 (0.31) | 0.07 (0.25) | 0.004 |
| **Manzanillo: Mean (SD)** | 0.14 (0.35) | 0.13 (0.33) | 0.15 (0.35) | 0.334 |
| **Puntarenas: Mean (SD)** | 0.11 (0.31) | 0.04 (0.21) | 0.13 (0.34) | <0.001 |
| **Tárcoles: Mean (SD)** | 0.04 (0.20) | 0.06 (0.23) | 0.04 (0.18) | 0.041 |
| **Recruitment fee: Don't know: Mean (SD)** | 0.00 (0.07) | 0.01 (0.09) | 0.00 (0.06) | 0.117 |
| **Recruitment fee: No: Mean (SD)** | 0.47 (0.50) | 0.44 (0.50) | 0.49 (0.50) | 0.056 |
| **No recruitment fee: Mean (SD)** | 0.49 (0.50) | 0.51 (0.50) | 0.49 (0.50) | 0.481 |
| **Recruitment fee was paid: Mean (SD)** | 0.03 (0.16) | 0.04 (0.21) | 0.02 (0.14) | 0.005 |
| **Subcontract: Don't know: Mean (SD)** | 0.00 (0.05) | 0.00 (0.07) | 0.00 (0.04) | 0.268 |
| **No Subcontract: Mean (SD)** | 0.59 (0.49) | 0.45 (0.50) | 0.64 (0.48) | <0.001 |
| **Subcontract: Yes: Mean (SD)** | 0.35 (0.48) | 0.39 (0.49) | 0.34 (0.47) | 0.054 |
